# microRNA Biomarkers in Clinical Study

**DOI:** 10.3390/biom11121810

**Published:** 2021-12-02

**Authors:** Hsiuying Wang, Yi-Hau Chen

**Affiliations:** 1 Institute of Statistics, National Yang Ming Chiao Tung University, Hsinchu 30010, Taiwan; 2 Institute of Statistics, Academia Sinica, Taipei 11529, Taiwan; yhchen@stat.sinica.edu.tw

MicroRNAs (miRNAs), short non-coding RNAs, are involved in the initiation and progression of many human diseases that also play a key role in immune response and drug metabolism modulation. In recent years, miRNA functions have been examined in vitro and in vivo model systems, and miRNA biomarkers, miRNA regulatory networks, and miRNA pathways have been identified. miRNAs can be detected in almost body fluids such as blood. For the easy availability, differences in the levels of circulating miRNAs can effectively help diagnose disease, prognosticate clinical outcome, and contribute to interindividual variability in treatment response. miRNAs have been linked to many human diseases, including various cancers, digestive diseases, and neurological diseases [1,2,3]. In addition, molecular biomarkers could offer an objective and quantitative measurement of disease states. Therefore, the diagnosis using miRNA profiling and the treatment targeting specific miRNAs are useful to develop personalized medicine in the future.

This Special Issue published seven papers, including five original research articles and two reviews covering interesting miRNA biomarker studies for different diseases including cancers and bone diseases.

Platelet concentrate (PC) transfusions are widely used to save the lives of patients with acute blood loss. This blood component needs special storage in blood banks. Due to the possible risk of bacterial contamination, a longer platelet storage duration has not been recommended. Therefore, it is essential to screen molecular biomarkers to assess and monitor the physiological viability of platelets in PC. Maués et al. [4] used an approach to identify miRNAs in normal human platelet sRNA-Seq data from the GSE61856 repository. A new collection of miRNAs (miR-486-5p, miR-92a-3p, miR-103a-3p, miR-151a-3p, miR-181a-5p, and miR-221-3p) was identified by their study that showed a sensitivity expression pattern due to biological platelet changes during storage. These miRNAs could be used as potential biomarkers to measure the quality and viability of the PC during storage. 

Inflammatory breast cancer (IBC) is a rare but aggressive variant of breast cancer with a poor prognosis. Fahim et al. [5] profiled the dysregulated expression of miRNAs in primary samples of IBC and non-IBC tumors using human breast cancer miRNA PCR array and identified 28 dysregulated miRNAs in IBC compared with non-IBC tumors group. In addition, Ozawa et al. [6] indicated the relevance of four miRNAs derived from extracellular vesicles as diagnostic markers in breast cancer (BC). 

Colorectal cancer (CRC) is the third leading cause of cancer death in the world, and miRNA played an important role in the promotion or inhibition of apoptosis in CRC [7]. Duran-Sanchon et al. [8] validated the previously suggested miR-1228-3p as an adequate endogenous control for circulating miRNA analysis in CRC by comparing its suitability with the widely used miR-16-5p, and they also evaluated the influence of hemolysis on both miRNAs. Human papillomavirus (HPV) infects millions of people each year, but the majority of individuals infected with HPV do not exhibit any clinical symptoms. Dysregulated miRNA expression has been identified to play a role in the context of high-risk HPV infection. Al-Eitan et al. [9] investigated the miRNA expression profiles of warts induced by low-risk HPV, and discovered that miR-27b, miR-24-1, miR-3654, miR-647, and miR-1914 were downregulated while miR-612 was upregulated in warts compared to normal skin.

The two review papers are related to bone diseases. Osteoporosis is one of the leading causes of bone fractures. miRNAs have been shown to be associated with the development of osteoporosis and bone fracture risk. Donati et al. [10] provided an overview of circulating miRNAs that were suggested as promising biomarkers for osteoporosis, which could be helpful for early diagnosis, monitoring of treatment response, and assessing the fracture risk in osteoporotic patients. In addition to osteoporosis, osteoarthritis is also an age-related pathology that contributes to bone fractures. Bottani et al. [11] reviewed circulating miRNA biomarkers for evaluating predisposition, diagnosis, and prognosis of osteoporosis, bone fracture/fragility, and osteoarthritis. These two review papers revealed the important role of miRNAs in bone diseases.

This Special Issue covers several diseases that miRNAs can be served as valuable clinical biomarkers. The seven papers published in this Special Issue provide significant research or review works for understanding the dysregulation of miRNA in these diseases, which, in turn, improve understanding of the potential of miRNA therapeutics.
